# Yes-Associated Protein Expression in Head and Neck Squamous Cell Carcinoma Nodal Metastasis

**DOI:** 10.1371/journal.pone.0027529

**Published:** 2011-11-09

**Authors:** Lin Ge, Matthew Smail, Wenxia Meng, Yu Shyr, Fei Ye, Kang-Hsien Fan, Xiaohong Li, Hong-Mei Zhou, Neil A. Bhowmick

**Affiliations:** 1 Department of Medicine, Cedars-Sinai Medical Center, Los Angeles, California, United States of America; 2 State Key Laboratory of Oral Diseases, Sichuan University, Chengdu, Sichuan, China; 3 Department of Biostatistics, Vanderbilt University, Nashville, Tennessee, United States of America; 4 Department of Cancer Biology, Vanderbilt University, Nashville, Tennessee, United States of America; 5 Department of Oral Medicine, West China Hospital of Stomatology, Sichuan University, Chengdu, Sichuan, China; Karolinska Institutet, Sweden

## Abstract

**Introduction:**

Yes-associated protein (YAP) is considered an oncogene found amplified in multiple tumors, including head and neck squamous cell carcinoma (HNSCC). However, the role for YAP expression in HNSCC is not understood. Based on the central role of YAP in the hippo pathway, we tested if YAP was associated with the stage of HNSCC progression and metastatic potential.

**Methods:**

To determine the expression of YAP in human benign and HNSCC tissue specimens, immunohistochemical analyses were performed in whole tissue samples and tissue microarrays. The expression of YAP in tissues of microarray was first associated with clinic-pathologic factors and results verified in samples from whole tissue sections. To investigate the role of YAP and p63 in regulating HNSCC epithelial to mesenchymal transition, epithelial and mesenchymal markers were assayed in Fadu and SCC-25 cells, HNSCC cells with endogenously elevated YAP expression and siRNA-mediated expression knockdown.

**Results:**

Analysis of human HNSCC tissues suggested YAP expression was elevated in tumors compared to benign tissues and specifically localized at the tumor invasive front (p value <0.05). But, indexed YAP expression was lower with greater tumor grade (p value  = 0.02). In contrast, p63 expression was primarily elevated in high-grade tumors. Interestingly, both YAP and p63 was strongly expressed at the tumor invasive front and in metastatic HNSCC. Strikingly, we demonstrated YAP expression in the primary HNSCC tumor was associated with nodal metastasis in univariate analysis (p value  = 0.02). However, the knockdown of YAP in Fadu and SCC-25 cell lines was not associated with changes in epithelial to mesenchymal transdifferentiation or p63 expression.

**Conclusion:**

Together, YAP expression, in combination with p63 can facilitate identification of HNSCC tumors from hyperplastic and benign tissues and the metastatic function of YAP in HNSCC may not be a result of epithelia to mesenchymal transdifferentiation.

## Introduction

Head and neck squamous cell carcinoma (HNSCC) can involve oral cavity, larynx and laryngeal pharynx. Oral squamous cell carcinoma (OSCC) represents 90% of oral cancers. Alterations in the 11q22 amplicon is detected in 5–15% of OSCC [1]. The gene, Yes-associated protein (YAP), located in 11q22, is specifically amplified in 4/23 of OSCC [2]. YAP was initially proposed as an oncogene following the identification of its expression in mouse tumor models [3], [4]. Later, multiple groups reported the up regulation of YAP in human cancers including those in the prostate, ovary, and colon [5], [6]. YAP is a critical transcription factor in the Hippo signaling pathway, initially identified for sensing and regulating organ size [7]. As such, YAP disregulation was further proposed as candidate oncogene in hepatocellular carcinoma, non-small-cell lung carcinoma, esophageal squamous cell carcinoma, ovarian cancer, gastric cancer [7]–[11]. However, in breast cancer, YAP is considered a tumor suppressor, based on reduced xenografted mammary tumor size in the context of YAP knockdown [8]. Opposing functions in different anatomical tissues might be due to its role with p53 family of proteins including p73 and p63, as well as interactions with transcription enhancer factor (TEF/TEAD) [12]–[17]. YAP is reported to cause EMT, stimulate proliferation, inhibit apoptosis, and promote tumor progression in a tissue-specific manner [7], [9]. The specific role of YAP in HNSCC is examined in this study.

A hallmark of HNSCC progression is elevated expression of the protein p63. P63 has two mRNA products from independent promoters, with each mRNA having three splice variants giving altered C-termini. Of the six p63 isoforms, delta-Np63 alpha is dominant in high grade HNSCC [10], [11]. Recently YAP was reported to promote the degradation of delta-Np63 alpha in head and neck carcinoma cell lines [12]. Further, Np63 binds the YAP promoter and repress its expression [21]. Thus, an inverse correlation between Np63 and YAP was speculated in patient tissues. However, the inverse relationship between YAP and p63 expression was not supported in human HNSCC tissues or cell lines. Our data indicated that elevated YAP expression differentiates hyperplastic and low grade HNSCC from benign head and neck tissues, with an unexpected decrease in expression in high grade HSCC. However, there was consistent elevation of YAP expression localized at the HNSCC tumor invasive edge. Interestingly, YAP expression or its knockdown did not affect epithelial-mesenchymal transition (EMT) progression in HSCC cell lines, nor did it affect p63 expression. The role of YAP expression in differentiating benign head and neck tissue from HNSCC can support its use as a marker to complement p63 in identifying tumor margins.

## Methods

### Ethics Statement

The study was performed with the written approval of the Ethics Committee of West China College of Stomatology, Sichuan University. We acquired written consent from all patients. The use of the data from the tissues was considered exempt by the Institutional Review Board at Vanderbilt University.

### Patients and samples

Twenty-three OSCC patients and six oral leukoplakia patients confirmed by pathologic diagnosis werè included in this study. Among them, 22 primary tumor samples of OSCC had been obtained from 2004 to 2008, with a 34-month median follow-up period (25–73 month range). Six patients undergoing orthodontic surgery were involved as benign controls. Tissue samples from total thirty-five patients were collected during surgery or biopsy after obtaining informed consents from patients in West China Hospital of Stomatology. Samples were fixed in 10% neutralized formalin and then embedded in paraffin. Additionally, a tissue microarray (TMA) (“Oral squamous cancer tissue array with normal oral control tissue” from Imgenex, San Diego, CA, USA and “Multiple head and neck tumor with normal tissue array, with stage and grade info, 80 cases/80 cores “ from Biomax, Rockville, MD, USA) contained 128 tissue cores of benign, benign adjacent to tumor and squamous cell carcinoma tissues. With the exception of the benign samples, the clinical and pathological information of the malignant samples is arranged in Table 1.

**Table 1:** The source, clinical and pathological information of malignant samples.

		OSCC patients (22)	Imgenex (44)	Biomax (58)
**Organ**	Dermal	1	3	3
	upper aerodigestive	1	0	44
	Oral cavity	19	41	7
	Bone	1	0	4
**Sex**	Female	6	17	10
	Male	16	27	48
**Grade**	Poorly to moderately	8	36	35
	well	14	8	23
**Stage**	III–IV	12	N/A	38
	I–II	10	N/A	20
**Nodal metastasis**	Yes	4	N/A	18
	No	18	N/A	40
**Recurrence or metastasis**	Yes	5	N/A	N/A
	No	12	N/A	N/A
	missing	5	N/A	N/A

### Antibodies and immunohistochemistry

Paraffin-embedded tissue sections (5 µm) were deparaffinized and hydrated through xylene and graded alcohols using a standard protocol [13]–[15]. A volume of 1% antigen unmasking solution (Vector laboratories, Burlington, CA, USA) or Tris-EDTA buffer (10 mM Tris Base, 1 mM EDTA solution, pH 9.0) was used for antigen retrieval depending on the antibodies. Subsequent immunohistochemical staining used antibodies against YAP (1∶200, Santa Cruz Biotech, Santa Cruz, CA, USA) vimentin E-cadherin and p63 4A4 (1∶1000, Santa Cruz). Appropriate HRP-conjugated secondary antibodies and DAB incubation (Dako North America, Carpinteria, CA, USA) were used for visualization. The intensity of YAP staining was classified as negative (score 0), weak positive (score 1), positive (score 2), strong positive (score 3). The percentage of positive cells in total malignant cells was counted. Three (for TMA) or five (for paraffin blocks) independent fields were acquired for each section. The final index was obtained by multiplying the intensity with the percentage. The calculation of index for cytoplasmic and nuclear staining was performed independently. Since rare nuclear staining appears in TMA, oral benign lesions and normal tissues in paraffin blocks, the indexed YAP expression only stands for cytoplasmic expression. The staining of Np63 was recorded in percentage of positive cells.

### Cell culture, transfection and Western blot analysis

The Fadu cell line was kindly gifted by Dr. Yarbrough (Vanderbilt University). It was cultured in high glucose Dulbecco's Modified Eagle's Medium, supplemented with 10% fetal bovine serum and 0.1% penicillin/streptomycin. Fadu cells were transfected with YAP or scrambled siRNA (Santa Cruz Biotech). The cells were harvested 48 h following transfection and analyzed by Western blotting for YAP, p63, vimentin, E-cadherin or beta-actin (Santa Cruz). Respective horseradish peroxidase conjugated secondary antibodies (GE Healthcare, Piscataway, NJ, USA) were used to visualize the expression of proteins.

### Statistical analysis

YAP protein expression levels of the HNSCC tumor tissues were measured by cytoplasmic staining using the technique involved tissue microarray. Three data sets were first pooled together and a categorical variable called "type" was created (Table 1). "Type" refers to the data set that the observation comes from. It has three levels, "Imgenex", "Biomax" and "OSCC". For each variable, a linear model that includes this variable, type and variable-by-type interaction was fitted. If the interaction was not significant by analysis of variance (ANOVA), the interaction was removed from the data and a new linear model that included the variable and type was fitted again. YAP expression association with tumor grade, stage, metastasis status, and other patient variables were determined by univariate analysis. Of note, limited clinical information was available on the status of differentiation, stage, nodal metastasis. Np63 expression was correlated with indexed YAP staining using Pearson analysis. Descriptive statistics, including means, standard deviations, and ranges for continuous variables, as well as percent and frequencies for categorical variables, are presented in Table 2. In general, hypotheses were tested at the level of α = 0.05. Point estimates along with the corresponding p-values and 95% confidence intervals are reported (Table 2). Statistical analysis was carried out using R (Version 2.10.1).

**Table 2:** Regression analysis for YAP cytoplasmic staining.

variables	Observations	univariate		
		Coefficient estimate	(95% confidence interval)	p-value
Age	124			
>60 y vs. ≤60 y		0.12	(−.29,.52)	0.85
Sex	124			
female vs. male		12.84	(−18.42,44.10)	0.42
Grade	124			
poorly vs. well differentiated		−34.81	(−63.934, −5.685)	0.02
Stage	80			
III–IV vs. I–II		10.31	(−22.51,43.13)	0.53
Nodal metastasis	80			
yes vs. no		41.66	(7.15,76.17)	0.02
Recurrence or Metastasis	22			
yes vs. no		37.18	(−25.041, 99.407)	0.23
missing vs. no		3.98	(−58.241,66.207)	0.9

## Results

### YAP expression levels correlate with head and neck squamous cell carcinoma and pathologic grade

**Figure 1:**
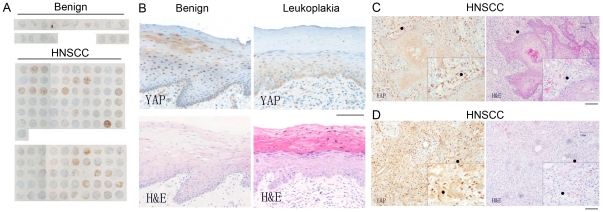
YAP protein expression was localized by immunohistochemistry in head and neck squamous cell carcinoma (HNSCC). (A) Tissue microarray data suggested YAP was up-regulated in HNSCC compared with benign tissue and benign tissue adjacent to tumor. (B) YAP was weakly expressed in the cytoplasm of basal cells, parabasal cells and suprabasal cells. Occasional expression of YAP in the nucleus of suprabasal cells in oral benign tissue and oral leukoplakia tissue was detected. (C) YAP expression was localized in the nucleus of stromal fibroblasts and (D) the cytoplasm of endothelial cells, as well as both cytoplasm and nucleus of HNSCC epithelia.

**Figure 2:**
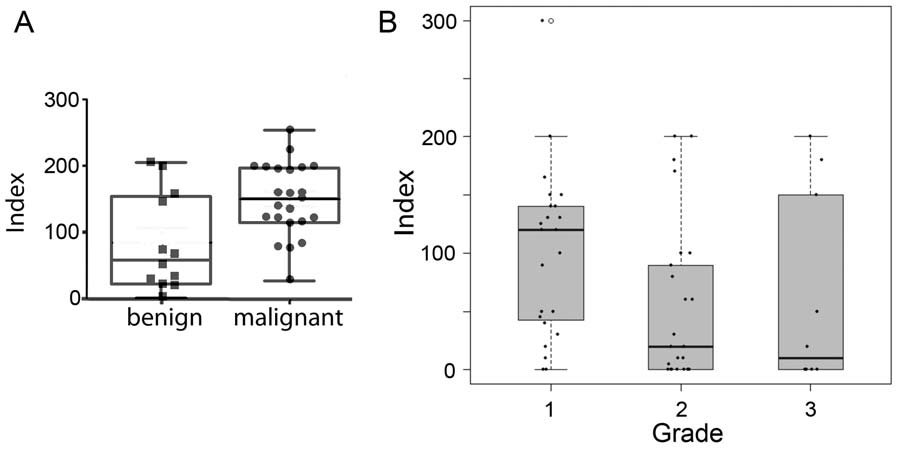
Indexed YAP expression in benign and HNSCC. (A) Malignant tissues presented a significantly greater indexed YAP staining compared to benign tissues (N = 35, P<0.05). (B) YAP expression, indexed by staining intensity, was correlated to HNSCC grades 1–3 (N = 80). The elevated YAP expression in Grade 1 HNSCC was significantly greater than that found in grades 2 and 3. There was no statistical difference between the expression of YAP in grades 2 and 3.

YAP expression in the tissue arrays of HNSCC and benign samples were analyzed by immunohistochemistry. HNSCC had high YAP expression compared to oral benign tissue or benign tissue adjacent to tumor. YAP expression was further localized in cancer and specifically elevated at the tumor borders (Figure 1). Additionally oral leukoplakia tissues from paraffin blocks had similar expression to oral benign tissues, where epithelium of the suprabasal layer had both cytoplasmic and nuclear YAP localization. The basal and parabasal cells of oral leukoplakia had primarily cytoplasmic YAP expression. The TMA of independent patients illustrated dramatic differences between malignant and benign tissues, where YAP staining of benign tissues in TMA were near undetectable. The quantitative data of indexed YAP expression illustrated in Figure 2 are derived from paraffin sections of whole tissues, as these can be best assessed pathologically for tumor margins (n = 35, p value ≤0.05). Careful analysis of whole tumor tissue sections revealed that the stromal compartment containing fibroblasts and endothelial cells in the benign tissues did not express YAP. However, the stromal compartment adjacent to carcinoma tissue generally had up-regulated YAP expression. The tissue and cellular distribution pattern of YAP expression interestingly associated with cancer status of the epithelia.

**Figure 3:**
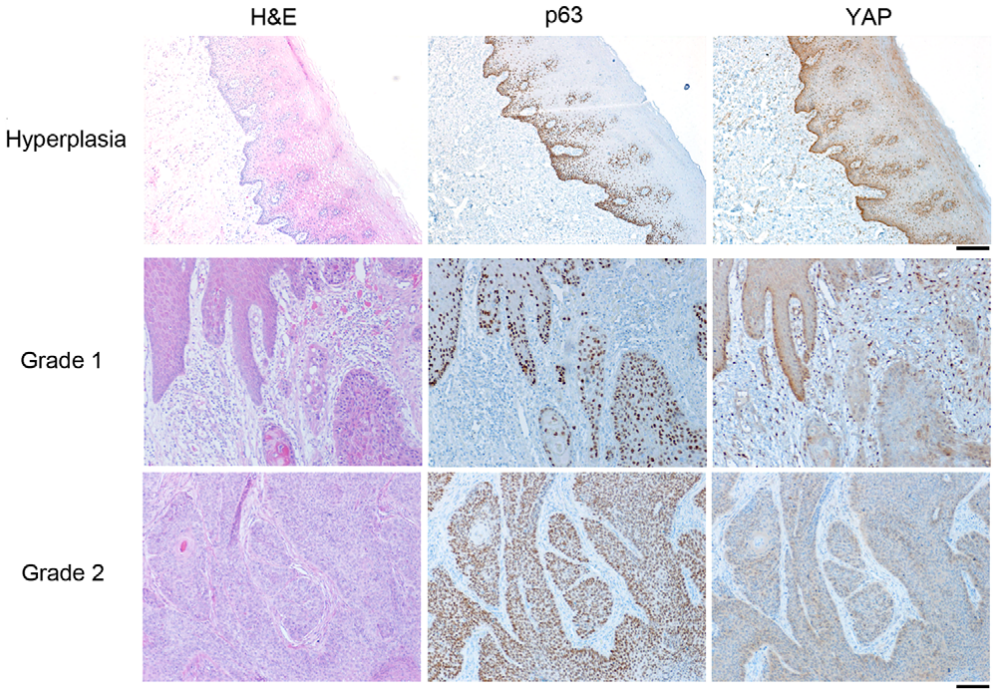
Correlation between YAP and p63 expression was identified in hyperplastic, grade 1, and grade 2 HNSCC. In hyperplastic tissue, p63 was expressed in basal and para-basal cells, whereas YAP was localized primarily in basal compartment. In grade 1 HNSCC, the number of cells expressing p63 was found in approximately 50% of the epithelial cell and YAP was strongly expressed in the basal layer with significant added expression in the stromal compartment. For grade 2 HNSCC, p63 was expressed in nearly all the malignant epithelial cells with significant decrease YAP expression.

**Figure 4:**
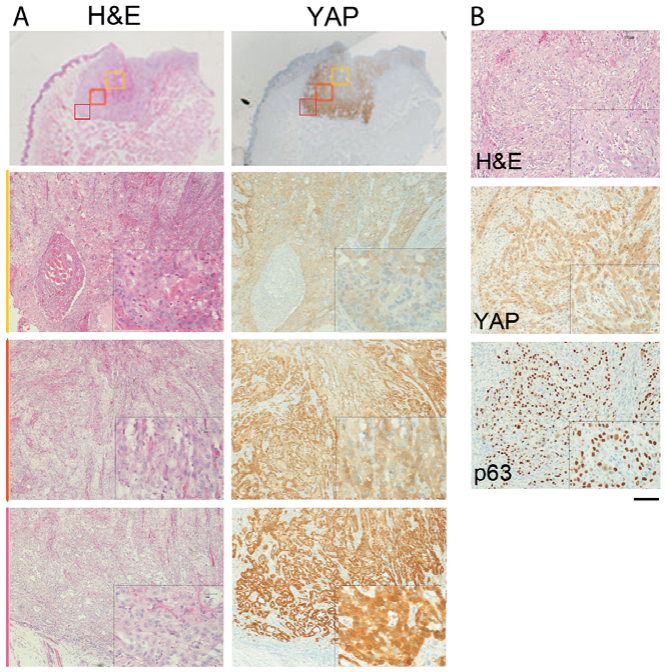
YAP expression in the HNSCC tumor invasive front is up regulated. (A) YAP expression was up regulated in the cytoplasm as well as expressed in the nucleus at the tumor edge. The colored boxes (yellow, orange, red) in the top panels are expanded in the corresponding panels below according to the respective colored lines on the left. This was done to specifically illustrate the differences in YAP expression at the distal tumor margins versus the proximal central region. (B) Malignant cells directly within the invasive front specifically had high nuclear YAP expression and high p63 expression. The scale bar represents 200 µm and 25 µm in the primary and inset images, respectively.

The expression of YAP was next correlated to clinical information of tumor progression. Univariate analysis indicated a significant difference in YAP expression with respect to the variables of differentiation and nodal metastasis, as opposed to tumor grade (Table 2). So, the result from a larger sample set demonstrated that YAP cytoplasmic expression increased in well-differentiated squamous cell carcinoma compared with poorly differentiated (p value  = 0.02). However, the data from 80 patients, suggested that nodal metastasis was associated with up regulation of YAP cytoplasmic expression (p value  = 0.02). Other covariates such as age, sex, organ site, and stage did not have differences in YAP expression (Table 2). Based on known expression of delta-Np63 alpha in high grade HNSCC [10], [11] and recent reports suggesting YAP-mediated degradation of delta-Np63 alpha [12], we tested the presence of an in vivo correlation of YAP expression and delta-Np63 alpha in serial sections from paraffin blocks. We observed the intensity of YAP decreases with elevated p63 expression and higher grade (Figures 3 and 4).

### The expression of YAP and invasive progression of head and neck squamous cell carcinoma

**Figure 5:**
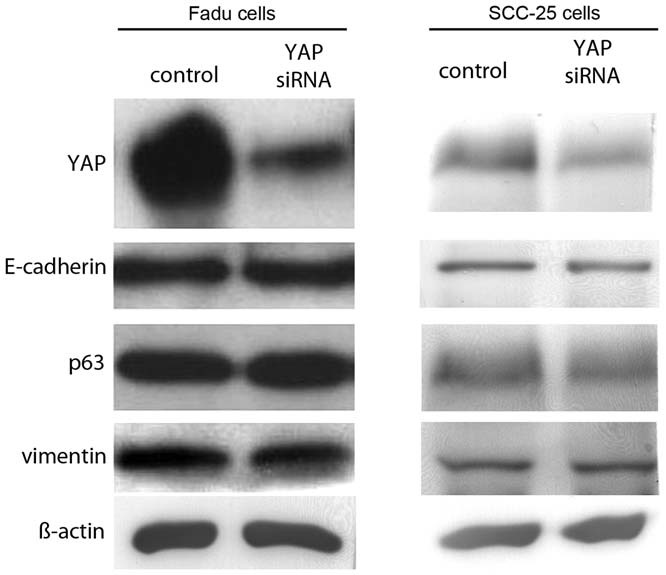
The YAP expression was not associated with p63 and EMT marker regulation in Fadu and SCC-25 cells. Western blot of (A) Fadu and (B) SCC-25 cells in control and those with YAP knocked by siRNA, had similar expression levels of p63, vimentin and E-cadherin. Beta-actin was used as a loading control.

Most strikingly, we observed differences in YAP expression within the tumor, where the margins of frank tumor had strong YAP expression compared to proximal areas (Figure 5). Interestingly, HNSCC areas of invasion had both strong YAP and p63 expression compared to the body of the tumor. While, p63 was localized exclusively in the nucleus, YAP epithelial expression was observed in both nuclear and cytoplasmic compartments. This observation was consistent with the observation in cell culture where YAP expression was elevated in cells on the edge or cells in small clusters [6]. But, the parallel correlation of p63 and YAP expression has not been made previously.

**Figure 6:**
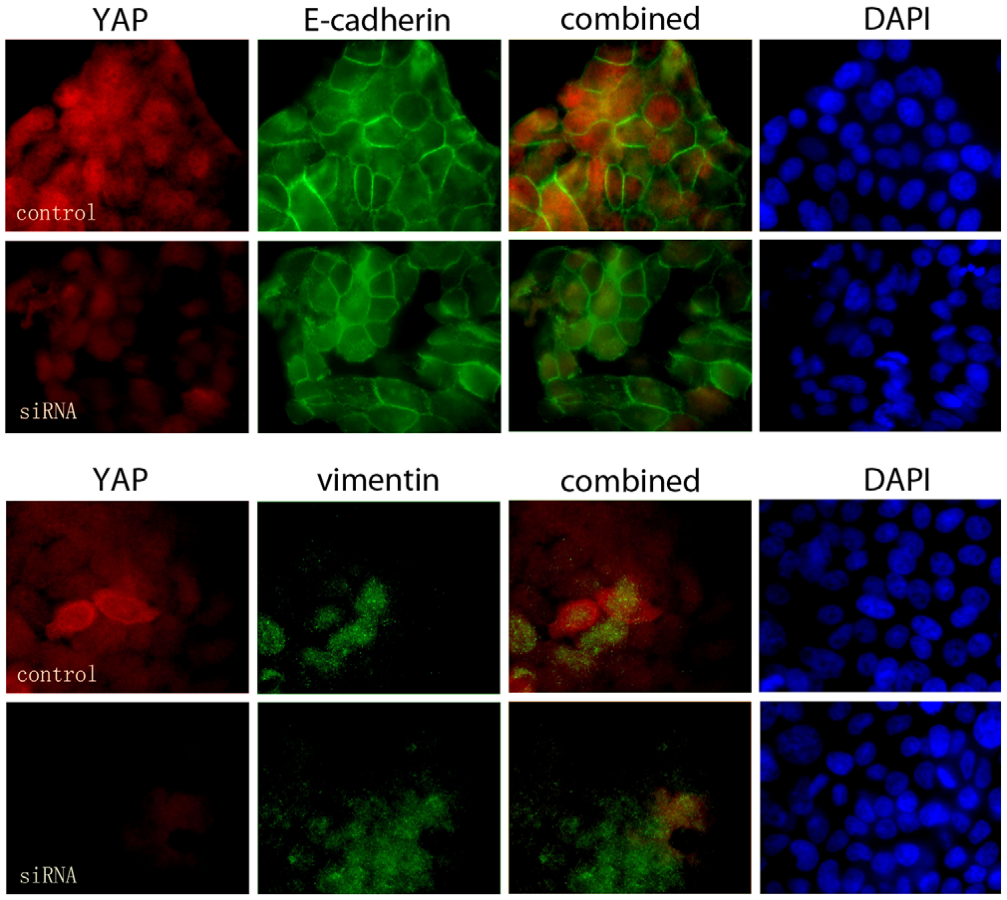
YAP expression does not affect E-cadherin and vimentin localization in the Fadu human HNSCC cell line. Immunofluorescent localization of E-cadherein, and vimentin was performed in control and YAP siRNA knocked down Fadu cells.

**Figure 7:**
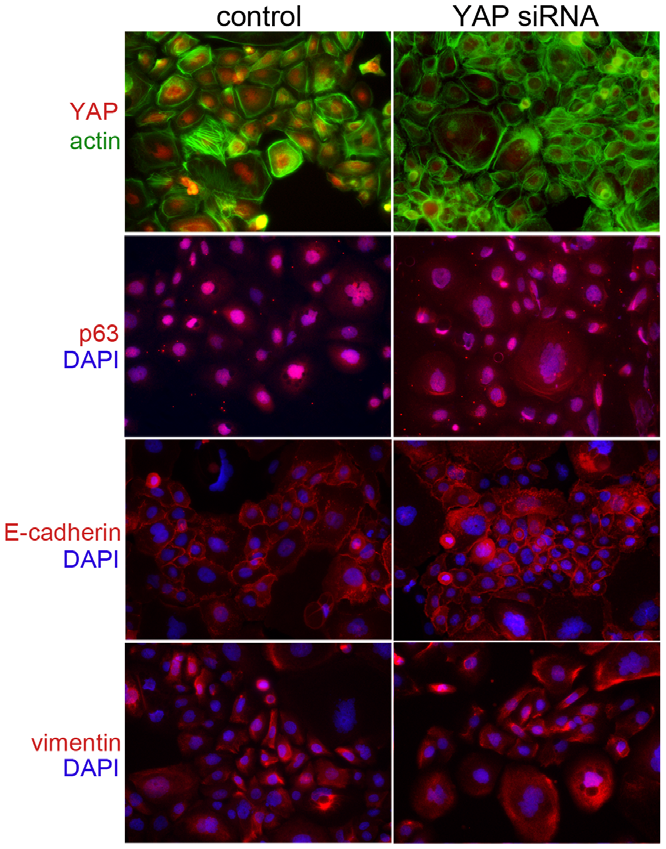
YAP expression does not affect p63 and EMT marker localization in the SCC-25 human HNSCC cell line. Immunofluorescent localization of actin, p63, E-cadherein, and vimentin was in SCC-25 following YAP knockdown was similar to that of control. The red YAP staining is counter stained with green actin localization. The blue nuclear Hoechst counter stain was used for the red p63, E-cadherein, and vimentin. The color of the text next to the panels represent the respective staining.

The relevance of elevated YAP expression at the tumor margins was rationalized to be potentially associated with EMT, as it has been in the past. Further correlation of YAP to p63 expression was tested in two established HNSCC cell lines. To determine if YAP was associated with EMT progression in head and neck cancer, Western blots for Fadu and SCC-25 human HNSCC cells were performed. We found that both had endogenously elevated YAP expression, with Fadu cells having significantly greater expression compared to the SCC-25 (Figure 5). P63 was expressed similarly in both lines. Interestingly, Fadu and SCC-25 cells expressed both the epithelial marker, E-cadherin, and mesenchymal marker, vimentin. To determine the impact of YAP expression on p63 and EMT markers, we knocked down YAP expression by siRNA. Western blotting and used immunofluorescence to localize the expression of the proteins in the context of YAP knockdown (Figures 5, 6, 7). However, the knockdown of YAP did not change p63 expression. Despite previous reports of the role of YAP in EMT, there were little changes in expression of EMT associated proteins, E-cadherin or vimentin, in independent Western blot experiments. Figures 6 and 7 illustrated negligible changes in immune-localization of p63, E-cadherin, and vimentin expression in Fadu and SCC-52 cells in control and YAP knocked down conditions. The added co-staining for F-actin enabled visualization of the maintenance of cortical actin structures in the both control and YAP knockdown conditions (Figure 7). Thus, the clear differential expression of YAP in metastatic and non-metastatic HNSCC patient tissues and interesting expression at the tumor margins was likely not a result of its role in EMT. Together, the data supports an alternative role for YAP expression in HNSCC and its association with greater nodal metastatic progression.

## Discussion

The study was focused on YAP expression patterns in head and neck squamous cell carcinoma and oral squamous cell carcinoma. Indexed YAP expression correlated with HNSCC tissue grade, site, and nodal metastasis through univariate analysis. Similarly, the analysis of OSCC tissues indicated a significant association of indexed YAP cytoplasmic expression with nodal metastasis. The clinical indicators, organ, differentiation grade and nodal metastasis were significantly correlated to the index of YAP staining. The index of YAP expression was elevated in well-differentiated HNSCC and diminished in higher grade HNSCC. This inverse correlation of YAP and HNSCC differentiation status differed from reports in liver cancer [16], but was similar to that found in breast cancer [7]. It implied the possible correlations between YAP cytoplasmic expression and these variables. But, regardless of HNSCC tumor grade, YAP expression was markedly higher at the tumor margins. Although we do not understand the apparent correlation of tumor differentiation status and YAP expression observed, the association with nodal metastasis implied the migration ability of YAP, consistent with previous reports [17]–[21]. In breast cancer, as well as HNSCC, YAP expression contributed to the migration ability of tumor cells [22], [23].

We sought to resolve this discrepancy in indexed YAP expression with decreasing stage yet association with metastasis. We observed YAP nuclear expression diminished with increasing clinical stage. However, cytoplasmic YAP indexed expression increased with incidence of HNCC nodal metastasis. A previous report of cytoplasmic YAP localization suggested a proliferative advantage in HNSCC [21]. The liver has elevated cytoplasmic YAP expression in poorly-differentiated carcinoma [24]. However, in benign tissues, YAP was more frequently expressed in the suprabasal cells as well as basal cells compared to parabasal cells, whose differentiation level lies between the former two types of cells. The results implied no correlation between p63, a basal cell marker, and YAP. However, we identified the inversed expression patterns of YAP and p63 in some specific phenotypes: 1) P63 was expressed in the parabasal cells of benign tissues in which YAP was not expressed (Figure 1) [25]. 2) P63 expression in poorly-differentiated HNSCC had little to no YAP expression (Figures 3 and 4). 3) Finally, YAP expression in benign suprabasal cells and well-differentiated HNSCC had little expression of p63 (Figure 3). The observed inverse expression of p63 and YAP was supported by the report that YAP expression mediates p63 degradation [12]. Yet, at the tumor's invasive front, p63 and YAP were both expressed.

The specific localization of YAP in cells at the tumor margins (Figure 4) and association with nodal metastasis initiated our studies on its role in metastatic progression. Based on mechanisms of metastatic progression that include individual cell migration and collective cell movement, we tested if YAP expression correlated with EMT or contact inhibition, respectively. Recently the ligand of epidermal growth factor receptor, amphiregulin, was reported to contribute to the proliferation and migration function of YAP in MCF10A [26]. YAP over expression in MCF10A, a breast non-transform cell line, can cause EMT [3], [27]. Thus far, the role of YAP in EMT has been carried out in MCF10A, which does not endogenously express YAP. Thus, we used Fadu and SCC-25 cells, common HNSCC cell lines that expresses YAP. However, traditional markers of EMT progression were not altered by YAP knockdown. (Figures 6, 7). Interestingly, in cell culture, greater YAP expression was found on the edges of cell clusters [6]. The contact inhibition function was first described in organ size control research. The over expression of YAP lead to liver size expansion, resulted from unrestricted cell proliferation [28], [29]. Thus, YAP expression in HNSCC may function as a means of sensing tissue/tumor size at the leading edge of tumor margins.

The expression of YAP in carcinoma tissues, compared to benign or benign adjacent tissues was dramatic. Hyperplastic tissues, such as papillomas, were found to have elevated YAP expression. In further examination of YAP expression in leukoplakia, a precancerous disease with a 10% chance to become OSCC, we found it was similar to benign tissue (Figure 1). The concept of field cancerization, was initially proposed in HNSCC, to describe changes in epithelial cells adjacent to the cancer. Some of these changes in the non-transformed cells were associated with genetic alteration and could not be considered benign tissue. We found YAP was over-expressed in malignant cells of HNSCC as well as associated endothelia and fibroblasts. Similar YAP expression was not observed in parallel benign and precancerous tissues. On the whole expression levels of benign tissues or hyperplastic tissues appeared lower compared to that of carcinoma tissues. Thus, YAP expression can serve as a marker to distinguish pre-malignant and malignant progression of HNSCC in tissue sections that may have otherwise ambiguous tumor margins. Currently, histologic cues and p63 expression help determine margins of HNSCC. The additional use of YAP expression may benefit the verification of histological low-grade HNSCC.
